# Diagnosis and management of brain metastasis from thyroid cancer

**DOI:** 10.1530/EO-25-0002

**Published:** 2025-05-16

**Authors:** Vincent Cascio, W Reed Doerfler, Charit Taneja

**Affiliations:** ^1^ Division of Endocrinology, Diabetes, and Metabolism, Donald and Barbara Zucker School of Medicine at Hofstra/Northwell, North Shore University Hospital, Manhasset, New York, USA; ^2^ University of Pittsburgh Medical Center, Pittsburgh, Pennsylvania, USA

**Keywords:** thyroid cancer, brain metastasis, stereotactic radiosurgery, targeted therapy, radioactive iodine

## Abstract

The brain is an uncommon location for metastatic spread from thyroid cancer. Given the rarity of the condition, the data regarding various management options for such patients are suboptimal. Radioactive iodine is seldom useful for brain metastases owing to variable uptake and unclear benefit. Surgical resection and stereotactic radiation remain the first-line treatment options for a limited number of brain metastases from thyroid cancer, as they not only provide local control and symptomatic relief but can also improve survival. Whole-brain radiation therapy has been used for patients with multiple brain metastases but has largely fallen out of favor due to the availability of more targeted and tolerable options. Systemic therapy with kinase inhibitors is a novel and promising area of research in this field, with an increased utilization of molecular testing to identify targetable mutations. Treatment plans for patients with brain metastases from thyroid cancer should be highly individualized and tailored to the specific patient by a multidisciplinary care team.

## Introduction

The incidence of thyroid cancer has risen considerably over the past several decades, likely due to the widespread use of ultrasound leading to increased detection of small, indolent tumors, and increased use of fine-needle aspiration for smaller nodules (Megwalu & Moon 2022). Although recent studies suggest a slight decline in overall incidence since 2014, there seems to have been a continued increase in the incidence of larger tumors and incidence-based mortality (Megwalu & Moon 2022). Nevertheless, thyroid cancer is still associated with excellent outcomes, with an overall 5-year survival rate of 99% (Siegel *et al.* 2021). Tumors with more aggressive histology and metastatic disease are associated with significantly poorer outcomes. Brain metastasis from thyroid cancer is rare, occurring in less than 2% of total cases, but is often associated with a grim prognosis (Chiu *et al.* 1997, Barnholtz-Sloan *et al.* 2004, Henriques de Figueiredo *et al.* 2014). The burden of brain metastasis from thyroid cancer could increase in the future due to the increasing incidence of larger, more aggressive tumors, increased utilization of imaging, and an aging society. We aim to review and highlight the current literature on brain metastasis from thyroid cancer, focusing on diagnosis and treatment options while addressing current gaps in the research and potential developments in the field.

## Epidemiology and risk factors

Thyroid cancer rarely metastasizes to the brain, occurring in only 0.15–1.4% of patients with differentiated thyroid cancer (DTC), though data from postmortem evaluations suggest that brain metastasis may be underdiagnosed (Gomes-Lima *et al.* 2019). Metastasis to the brain is more common in certain histological variants of thyroid cancer, including follicular, oncocytic (Hürthle cell), and poorly differentiated thyroid cancer (Chiu *et al.* 1997, Henriques de Figueiredo *et al.* 2014, Gomes-Lima *et al.* 2019, Osborne *et al.* 2019, Li *et al.* 2020). More aggressive subtypes of papillary thyroid carcinoma (PTC) such as columnar, solid, hobnail, and tall cell are also implicated (Gubbiotti & Livolsi 2023). Brain metastasis is also seen in both medullary thyroid cancer (MTC) and anaplastic thyroid cancer (ATC), though the prevalence is difficult to estimate due to the rarity of these cancers (Chiu *et al.* 1997, Kim *et al.* 2021, Lee *et al.* 2022, Wolff *et al.* 2023, Wu *et al.* 2023). Brain metastasis is more common in thyroid cancer patients with other distant metastases, large primary tumors, and extrathyroidal invasion of the primary tumor (Dinneen *et al.* 1995, Chiu *et al.* 1997). Early and late molecular driver events including *BRAF^V600E^*, *TERT*, and *TP53* mutations are associated with more advanced, de-differentiated tumors and aggressive disease. Combinations of these mutations are among those commonly seen in patients with brain metastasis from thyroid cancer (Osborne *et al.* 2019).

## Diagnosis and prognosis

Thyroid cancer typically metastasizes to the lung and bone, and almost all patients with brain metastases from thyroid cancer have additional metastases to these sites (Gomes-Lima *et al.* 2019, Kim *et al.* 2021). The median interval time from initial diagnosis of thyroid cancer to diagnosis of brain metastasis is 3–10 years (Chiu *et al.* 1997, Choi *et al.* 2016, Gomes-Lima *et al.* 2019, Gubbiotti & Livolsi 2023). Given its highly aggressive nature, ATC is associated with a significantly shorter median interval of less than 1 year from diagnosis of thyroid cancer to diagnosis of brain metastasis (Chiu *et al.* 1997, Salvati *et al.* 2001).

Most patients with brain metastasis from thyroid cancer will experience neurologic symptoms (Chiu *et al.* 1997, Kim *et al.* 2021). The most common symptom is headache, and other symptoms that have been reported include nausea, polyuria, focal motor and sensory deficits, ataxia, vision loss, and seizure (Chiu *et al.* 1997, Gomes-Lima *et al.* 2019).

The usual prognosis for thyroid cancer is generally positive, with 10-year survival estimates of greater than 90% for DTC and 70–90% for MTC (Park *et al.* 2021). In contrast, ATC is one of the most aggressive cancers, with 1-year survival of only 20% and disease-specific mortality of almost 100% (Lee *et al.* 2022, Limaiem *et al.* 2024). Brain metastases are associated with a grim prognosis in all forms of cancer, which is also true for thyroid cancer. Overall survival of patients with brain metastases from thyroid cancer ranges from 4 to 33 months, with survival at 5 years around 10% (Chiu *et al.* 1997, McWilliams *et al.* 2003, Henriques de Figueiredo *et al.* 2014, Choi *et al.* 2016, Saito *et al.* 2016). More aggressive histologic subtypes are associated with poorer survival (Gomes-Lima *et al.* 2019). Brain metastasis from ATC is associated with a median survival of only 1 month, compared to 9 months for patients with ATC without brain metastasis (Chiu *et al.* 1997, Salvati *et al.* 2001, Lee *et al.* 2022).

Poor prognostic factors for patients with brain metastasis from thyroid cancer include an increasing number of brain metastases, coexisting lung metastases, and poor functional status (Chiu *et al.* 1997, Henriques de Figueiredo *et al.* 2014, Choi *et al.* 2016, Saito *et al.* 2016, Yoo *et al.* 2022). Initial treatments, including RAI and thyroidectomy, do not affect survival once brain metastases are diagnosed (Chiu *et al.* 1997). Importantly, most patients die from extracranial disease rather than from the brain metastases (McWilliams *et al.* 2003).

## Imaging recommendations

MRI is more sensitive than CT for diagnosing metastatic brain lesions and is the imaging modality of choice. FDG-PET scans are less sensitive than other cross-sectional modalities and may underdiagnose brain lesions given the physiologic glucose uptake of the brain (Salvati *et al.* 2001).

The American Thyroid Association (ATA) guidelines suggest that MRI of the brain should be considered in high-risk patients with DTC who have elevated serum thyroglobulin (generally >10 ng/mL) after total thyroidectomy, negative neck and chest imaging, and neurologic symptoms. It can also be considered in such patients before TSH-stimulated RAI therapy, as these patients may be at risk for complications of tumor swelling (Haugen *et al.* 2016). Other sources suggest that imaging of the brain may be warranted in patients with DTC and any distant metastases, even if neurologic symptoms are not present (Gomes-Lima *et al.* 2019).

The ATA guidelines suggest that in patients with MTC, brain imaging should be performed in patients with metastatic disease and neurologic symptoms (Wells *et al.* 2015). In patients with ATC, brain imaging should be performed as a routine part of initial staging and if the patient develops neurologic symptoms (Bible *et al.* 2021).

## Considerations before initiating treatment

Many patients with metastatic DTC will have indolent disease that does not progress for many years. The initial treatment for DTC with distal metastasis is RAI. For stable RAI-resistant disease, observation may be appropriate, as systemic therapy is not curative and is associated with toxicities. These patients can often be monitored with serial clinical exams, imaging, and biochemical markers. Progressive or symptomatic disease usually requires treatment (Table 1). Unlike distant metastases to other organs, brain metastasis from thyroid cancer generally requires treatment due to the high associated morbidity and mortality.

**Table 1:** Summary of treatment options for brain metastases from thyroid cancer.

Treatment modality	Indications	Considerations
Surgery	• Single lesion • Large size • Neurologic symptoms	• Improved survival • Local control • Tumor debulking • Reduction of vasogenic edema • <10% risk of complications
SRS	• 2–4 lesions • Small size • No neurologic symptoms • Poor surgical candidate	• Possible survival benefit • Local control • Minimally invasive • Rapid recovery • 3% risk of radiation necrosis
WBRT	• >4 lesions • Unfavorable candidate for SRS • Life expectancy <3 months	• Decreased intracranial failure after surgical resection • Radiation-induced dementia • Pituitary dysfunction
RAI therapy	• Lesions concentrate iodine (rare)	• May also treat non-cerebral metastases • TSH-induced tumor growth, edema, and hemorrhage
Systemic therapy	• Advanced or rapidly progressive disease • Targetable mutation (e.g., BRAFV600E, RET, NTRK fusion)	• Minimal data in the setting of brain metastases • Non-curative • Systemic toxicity

SRS, stereotactic radiosurgery; WBRT, whole-brain radiation therapy; RAI, radioactive iodine; TSH, thyroid-stimulating hormone.

Before initiating treatment, it is important to estimate expected survival to help guide appropriate therapy. ECOG performance status less than 2 is the single most important prognostic factor for a good outcome after surgery or radiation (Oken *et al.* 1982, Henriques de Figueiredo *et al.* 2014). Other prognostic factors for successful treatment include age younger than 60 years, fewer than three brain metastases, and no extracranial metastasis before brain metastasis. Patients with multiple favorable prognostic factors may experience overall survival greater than 30 months with aggressive treatment (Choi *et al.* 2016).

Accurate subtyping of the primary tumor is important, as it may affect downstream treatment decisions. The histology of brain metastases is usually identical to that of the original tumor (Osborne *et al.* 2019). Mutational status is also increasingly relevant given the increasing availability of targeted therapies and should be evaluated in all primary tumors. This is particularly relevant for ATC, where the ATA guidelines emphasize that the immediate assessment of mutation status is crucial in these patients, as individualized targeted therapy is often the primary treatment (Bible *et al.* 2021).

## Surgery

The ATA guidelines for the treatment of DTC state that surgical resection and stereotactic radiosurgery are the mainstays of therapy for brain metastases (Haugen *et al.* 2016). Surgery provides numerous benefits, including local control, tumor debulking, reduction of vasogenic edema, relief of neurologic symptoms, and improved survival (Mut 2012, Carapella *et al.* 2018).

The survival benefit in patients treated with surgery is well documented. In an analysis of 47 patients with brain metastasis from thyroid cancer, Chiu *et al.* reported increased survival associated with surgery (16.7 vs 3.4 months). For patients with a single brain lesion, the survival benefit was even more pronounced (25.2 vs 2.4 months). Surgery was an independent predictor of better outcome and was the only therapy associated with a survival benefit (Chiu *et al.* 1997). More recent data have shown median survival of up to 30 months in those who are treated with surgery, compared to less than 12 months in those who are not (McWilliams *et al.* 2003, Henriques de Figueiredo *et al.* 2014, Choi *et al.* 2016, Gomes-Lima *et al.* 2019, Osborne *et al.* 2019). Aggregate data from case reports including 18 patients with brain metastasis from ATC demonstrate an overall survival of 9 months in those treated with surgery, compared to 1 month in those who were not, demonstrating a clear benefit even in patients with this aggressive form of thyroid cancer (McWilliams *et al.* 2003).

Surgery is associated with a less than 10% risk of major complications, which can include bleeding, infection, stroke, and neurological worsening. Patients with a single metastatic brain lesion should generally be offered surgery, particularly if the brain metastasis is large or if there are neurologic symptoms (Vogelbaum *et al.* 2022). Caution is advised in offering surgery to patients with multiple brain metastases, as the survival benefit is diminished and quality of life may be adversely affected.

## Stereotactic radiosurgery

Patients with brain metastasis from thyroid cancer are often poor candidates for surgery due to high rates of other distant metastases, poor performance status, and poor overall survival. Stereotactic radiosurgery (SRS) uses high doses of radiation targeted to the affected area while minimizing impact on surrounding healthy tissue. SRS is minimally invasive, with a short recovery time and a low rate of complications, including a 3% risk of radiation necrosis (Blomain *et al.* 2022, Byun *et al.* 2023). SRS is a viable alternative to surgery, and its use has increased over time, becoming the most used focal treatment modality for brain metastases (Mehta & Ahluwalia 2015, Blomain *et al.* 2022).

SRS is associated with 90% local control rates, although patient- and tumor-specific factors, including functional status, size and location of the lesions, and tumor histology, affect the response (Andrews *et al.* 2004, Gomes-Lima *et al.* 2019, Byun *et al.* 2023). The data on survival are not quite as clear. Gomes-Lima *et al.* evaluated 24 patients with brain metastasis from DTC and found a non-statistically significant trend toward improved survival associated with SRS (52.5 vs 6.7 months) (Gomes-Lima *et al.* 2019). Bernad *et al.* evaluated 23 patients with brain metastasis from thyroid cancer and also found a non-statistically significant trend toward improved survival associated with SRS (37.4 vs 12.3 months) (Bernad *et al.* 2010). Blomain *et al.* evaluated the outcomes of patients with brain metastasis from thyroid cancer who received radiotherapy, including 33 patients identified in their institution and 289 patients identified in the National Cancer Database (NCDB). They found a significant survival benefit associated with SRS when compared to whole-brain radiation therapy (WBRT) in the institutional cohort (10.7 vs 1.8 months), but this was not replicated in the NCDB cohort (Blomain *et al.* 2022).

SRS is generally offered to patients with a limited number of brain metastases, though there is evidence that SRS may also be a reasonable option in patients with more numerous lesions. A large-scale prospective observational study of patients with brain metastases from any malignancy showed no difference in overall survival in patients with 2–4 brain metastases compared to patients with 5 or more brain metastases treated with SRS alone (Yamamoto *et al.* 2014). The more important factor in determining patient outcomes may be the overall volume of metastases rather than the number of metastases (Mohammadi *et al.* 2012).

Currently there is no evidence to support the use of SRS over surgery. Studies comparing SRS to surgery for brain metastases from any malignancy have not found a significant survival difference, though data specific to thyroid cancer are lacking (Schöggl *et al.* 2000, O'Neill *et al.* 2003). SRS should be considered in patients who are poor surgical candidates, do not wish to pursue surgery, or present with advanced intracranial disease. The American Society of Clinical Oncology (ASCO) guidelines for the treatment of brain metastases from any malignancy state that SRS is a suitable therapy for patients with 1–4 unresected brain metastases. Caution should be advised in offering SRS for patients with poor performance status and no systemic therapy options, as they are not expected to derive a benefit from radiation therapy (Vogelbaum *et al.* 2022).

SRS of the resection cavity following surgery has been used to reduce local recurrence in a variety of cancers (Iwai *et al.* 2008, Limbrick *et al.* 2009). Adjuvant SRS is associated with decreased toxicity compared to adjuvant WBRT, but carries risks of radiation necrosis and leptomeningeal disease in certain cancers (Atalar *et al.* 2013). Neoadjuvant SRS has shown promise in minimizing these risks by allowing for tighter margins of radiation during treatment planning and “sterilizing” the tumor before manipulation, while offering similar local control rates (Rajkumar *et al.* 2022). Several phase 3 trials are underway comparing adjuvant to neoadjuvant SRS (Crompton *et al.* 2023).

## Whole-brain radiation therapy

WBRT has traditionally been a treatment for patients with multiple brain metastases, poor performance status, or life expectancy less than 3 months, mainly for palliative purposes. It has also been used as an adjuvant therapy with either surgery or SRS to reduce the rate of intracranial relapse and local recurrence (Patchell 2003, Brown *et al.* 2018).

In 1998, a randomized trial including 95 patients with brain metastases from any malignancy demonstrated that the addition of WBRT after complete tumor resection was associated with decreased intracranial failure and local recurrence, and a non-statistically significant trend toward improved survival (Patchell *et al.* 1998). Notably, this study included patients with well-controlled primary tumors and a single metastatic lesion to the brain, which is a rare find. WBRT after surgery or SRS has consistently failed to demonstrate a survival benefit in patients with brain metastasis from thyroid cancer (Chiu *et al.* 1997, McWilliams *et al.* 2003, Henriques de Figueiredo *et al.* 2014, Gomes-Lima *et al.* 2019).

Radiation-induced dementia needs to be considered, especially in those with longer life expectancy (Smith & Lee 2007). Chang *et al.* performed a randomized controlled trial comparing neurologic outcomes from SRS with adjuvant WBRT to SRS alone in patients with brain metastasis from any malignancy. The trial was stopped early due to a high probability of significant decline in learning and memory function by 4 months in the group receiving WBRT (Chang *et al.* 2009). There is evidence that treatment with memantine and the use of hippocampal avoidance may be effective at minimizing neurocognitive side effects (Brown *et al.* 2013, Brown *et al.* 2020).

Pituitary hormone dysfunction is a dose-dependent, long-term side effect of WBRT, and up to two-thirds of patients treated with cranial radiotherapy for intracerebral tumors may be affected (Appelman-Dijkstra *et al.* 2011, Scoccianti *et al.* 2015). Monitoring of pituitary hormones should be a routine part of follow-up care in all patients treated with WBRT, including assessment of thyroid function and the hypothalamic-pituitary-adrenal axis (Appelman-Dijkstra *et al.* 2011). Sparing of the hypothalamic-pituitary region during WBRT is a promising concept which requires further investigation (Mehta *et al.* 2020).

ASCO guidelines state that SRS, WBRT, and the combination of SRS plus WBRT are all reasonable options for more than 4 unresected or more than 2 resected metastases (Vogelbaum *et al.* 2022). However, due to the lack of survival benefit, concerns over neurotoxicity, and continuous improvements in SRS, the use of WBRT has largely fallen out of favor. Its use is mostly reserved for patients who have multiple brain metastases and are not deemed favorable SRS candidates (Mehta & Ahluwalia 2015).

## Radioactive iodine therapy

RAI is the standard of care in recurrent or metastatic DTC and has been shown to improve survival, though advanced DTC may lose the ability to concentrate iodine and thus become RAI-refractory (Durante *et al.* 2006, Mu *et al.* 2019). Iodine concentration by thyroid cancer brain metastases is variable, between 4 and 45% in small, retrospective studies, limiting its use in this setting (Chiu *et al.* 1997, Gomes-Lima *et al.* 2019, Osborne *et al.* 2019). Misaki *et al.* evaluated 167 patients with DTC who received RAI for metastases in the lung and/or bones, including nine patients who also had brain metastasis. None of the patients showed significant uptake in the brain despite accumulation in most extracerebral metastases (Misaki *et al.* 2000). A case series by McWilliams *et al.* included four patients with brain metastasis from thyroid cancer who received RAI, none of whom achieved an objective response (McWilliams *et al.* 2003). The ATA guidelines state that RAI can be considered for the treatment of brain metastases from DTC if the lesions concentrate iodine, but this is a weak recommendation based on low-quality evidence (Haugen *et al.* 2016).

Thyroid hormone withdrawal or administration of recombinant human thyrotropin (rhTSH) in preparation for RAI treatment is associated with a risk of neurologic complications due to TSH-induced tumor growth, cerebral edema, or sudden hemorrhage (Holmquest & Lake 1976, Datz 1986, Chiu *et al.* 1997). If RAI is planned in patients with known brain metastases, prior treatment with SRS and concomitant glucocorticoid therapy is recommended to minimize the risk of neurologic complications. The suggested steroid regimen is dexamethasone 2–4 mg every 8 h starting before rhTSH administration and tapered over a week after RAI therapy (Haugen *et al.* 2016). Similarly, dexamethasone 4–16 mg/day is recommended for ATC patients with neurologic signs or symptoms (Bible *et al.* 2021).

## Systemic therapy

Thyroid cancer often harbors mutations in *BRAF*, *RAS*, or *RET* oncogenes, as well as fusions involving receptor tyrosine kinases, which lead to tumor dedifferentiation and uncontrolled growth. These mutations present opportunities for targeted therapy in patients with metastatic thyroid cancer with advanced or rapidly progressive disease. There is minimal data on their effectiveness for brain metastasis from thyroid cancer, as most studies in this population have small sample sizes or were performed before the era of targeted therapy. Understanding the options for systemic therapy remains important as most patients with brain metastases will have other distant metastases as well.

Multikinase inhibitors (MKIs) work by disrupting multiple signaling pathways including *VEGFR*, causing inhibition of angiogenesis and disruption of tumor growth (Pitoia *et al.* 2022). FDA-approved MKIs for the treatment of thyroid cancer include vandetanib, cabozantinib, sorafenib, and lenvatinib. MKIs have been shown to improve progression-free survival and are used in RAI-resistant DTC, metastatic MTC, and ATC (Wells *et al.* 2012, Elisei *et al.* 2013, Zhang *et al.* 2023). It is not fully understood if MKIs cross the blood–brain barrier, but there is evidence of intracranial efficacy in other cancers which more commonly metastasize to the brain, including in RET-mutated lung cancer and renal cell cancer (Koutras *et al.* 2007, Lin *et al.* 2016, Drilon *et al.* 2018).

There have been case reports demonstrating stability and even partial intracranial response of brain metastases from thyroid cancer treated with MKIs (Shen *et al.* 2012, Pereira *et al.* 2024). In a phase 3 trial using lenvatinib in RAI-refractory thyroid cancer (SELECT), which included five patients with brain metastasis, the median duration of response in patients with brain metastasis was 9.3 months, compared to 30.5 months in patients without brain metastasis (Schlumberger *et al.* 2015, Gianoukakis *et al.* 2018). A retrospective study by Rendl *et al.* included five patients with brain metastasis from RAI-refractory DTC and noted a median overall survival of 12 months in patients treated with lenvatinib (Rendl *et al.* 2020). In a retrospective study including 24 patients with brain metastasis from DTC, 12 of whom were treated with MKIs, Gomes-Lima *et al.* found that use of MKIs was associated with improved survival (27.2 vs 4.7 months). The authors noted that the results should be interpreted with caution due to possible selection bias, as MKIs are not often used for patients with poor functional status (Gomes-Lima *et al.* 2019).

MKIs are not curative and are associated with considerable toxicities. Selective *RET* inhibitors, including selpercatinib and pralsetinib, are associated with significantly fewer toxicities and have shown promise in RET-mutated MTC and RET fusion-positive PTC (Wirth *et al.* 2020, Subbiah *et al.* 2021*b*). Intracranial efficacy for selpercatinib has been demonstrated in *RET*-mutated NSCLC (Subbiah *et al.* 2021*a*). A case report demonstrated good CSF penetration of pralsetinib, and it is expected to be an effective treatment for brain metastases from *RET*-mutated NSCLC (de Jong *et al.* 2023). There is minimal data on the effectiveness of these therapies on brain metastasis from thyroid cancer.

Dabrafenib/trametinib is a combination *BRAF*/*MEK* inhibitor shown to be effective in *BRAF^V600E^*-mutated ATC (Subbiah *et al.* 2018). There is some evidence of intracranial benefit of dabrafenib/trametinib for metastatic melanoma patients with brain metastases; however, there are currently no reports supporting this in patients with brain metastases from thyroid cancer (Davies *et al.* 2017). Larotrectinib and entrectinib are TRK inhibitors with effectiveness in *NTRK* fusion-positive thyroid cancers (Hong *et al.* 2020). Pitoia *et al.* reported the disappearance of multiple brain metastases in a patient with RAI-refractory DTC after treatment with larotrectinib (Pitoia 2021). Cytotoxic chemotherapy has not been found to benefit patients with brain metastases from thyroid cancer (McWilliams *et al.* 2003, Simões-Pereira *et al.* 2016). Research is underway evaluating the use of other mutation-specific therapies, small-molecule inhibitors to restore radioiodine avidity, radiopharmaceuticals, immunotherapy, and focused ultrasound for the treatment of metastatic thyroid cancer. Further studies are needed to evaluate the effectiveness of these novel therapies in the setting of brain metastasis.

## Conclusions

**Figure 1:**
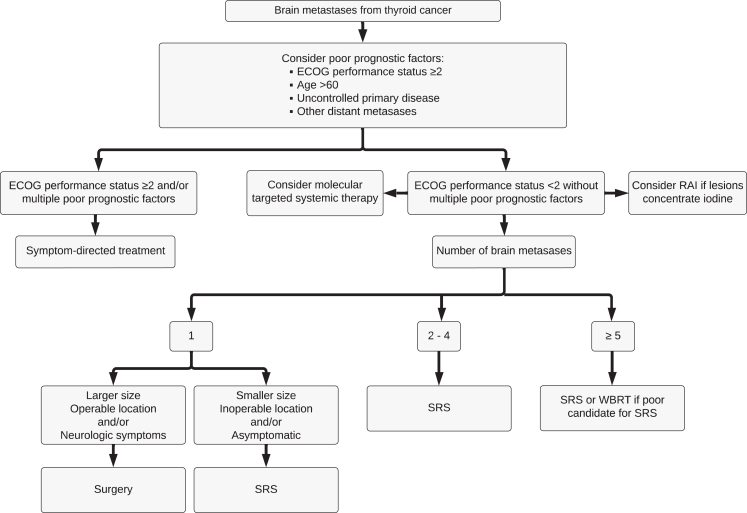
Proposed approach for the management of patients with brain metastases from thyroid cancer. ECOG, Eastern Cooperative Oncology Group; RAI, radioactive iodine; SRS, stereotactic radiosurgery; WBRT, whole-brain radiation therapy.

In summary, surgery and SRS are first-line treatments for brain metastases from thyroid cancer, as they provide local therapy, minimize symptoms, and potentially improve survival. Most patients should be offered either surgery or SRS regardless of the treatment being used for systemic disease. WBRT has fallen out of favor due to the lack of survival benefit and the risk of radiation-induced dementia. RAI therapy can be considered to treat lesions that concentrate iodine but poses a risk of complications from tumor swelling or hemorrhage. Although systemic therapy is traditionally reserved for patients who fail to respond to local therapy or have rapidly spreading or diffuse disease, recent data suggest good intracranial efficacy and a limited toxicity profile of highly targeted agents such as selpercatinib (in patients with RET mutations/fusions) and larotrectinib (in patients with NTRK fusions), which may support their earlier use in this small subset of patients. We have included a proposed approach to managing brain metastases from thyroid cancer (Fig. 1). Our suggested approach is similar to that highlighted by other experts in the field (Prinzi *et al.* 2024). The initial management should be based on evaluation of performance status and prognostic factors, with less aggressive approaches chosen for patients with multiple poor prognostic factors. Most data supporting survival benefit exist for neurosurgery and SRS, but systemic therapy has shown promise in recent reports and its full potential remains to be explored, especially considering the availability of highly targeted agents with good intracranial efficacy and improved tolerability.

Most studies evaluating brain metastasis from thyroid cancer include primarily patients with DTC, although many also incorporate smaller numbers of patients with MTC or ATC. The data are mostly limited to small, retrospective studies from single institutions with symptomatic patients. Potential biases in patient referral and treatment may limit their applicability. Unfortunately, given the rarity of this condition, prospective studies or randomized controlled trials are unlikely. Due to the suboptimal data available, individual treatment plans for patients with brain metastases from thyroid cancer should be highly tailored to each patient’s disease process.

## Declaration of interest

The authors declare that there is no conflict of interest that could be perceived as prejudicing the impartiality of this work.

## Funding

This work did not receive any specific grant from any funding agency in the public, commercial, or not-for-profit sector.

## Author contribution statement

VC contributed to conceptualization, literature review and analysis, writing – original draft, review and editing. WRD and CT contributed to conceptualization, literature review and analysis, writing – review and editing.
